# Apoptosis Induction by the Total Flavonoids from *Arachniodes exilis* in HepG2 Cells through Reactive Oxygen Species-Mediated Mitochondrial Dysfunction Involving MAPK Activation

**DOI:** 10.1155/2014/906941

**Published:** 2014-05-28

**Authors:** Huimin Li, Jing Chen, Chaomei Xiong, Han Wei, Changchang Yin, Jinlan Ruan

**Affiliations:** ^1^Key Laboratory of Natural Medicinal Chemistry and Resource Evaluation of Hubei Province, College of Pharmacy, Huazhong University of Science and Technology, No. 13 Hangkong Road, Wuhan, Hubei 430030, China; ^2^Basic Medical College of Jiujiang University, Jiujiang 332000, China

## Abstract

*Arachniodes exilis* is used as a folk medicine in China and proved to have antibacterial, anti-inflammatory, and sedative activities. In the present study, the antitumor effect of the total flavonoids of *A. exilis* (TFAE) against HepG2 cells was evaluated. The results showed that TFAE inhibited the growth of HepG2 cells in a dosage- and time-dependent manner. Flow cytometry and Hoechst 33342 fluorescence staining results showed that TFAE could significantly increase the apoptosis ratio of HepG2 cells, which is accompanied with increased intracellular reactive oxygen species (ROS) production and decreased mitochondrial membrane potential (ΔΨ*m*). Western blotting indicated that TFAE downregulated the ratio of Bcl-2/Bax, increased cytochrome c release, and activated the caspases-3 and -9. Further analysis showed that TFAE stimulated the mitogen-activated protein kinase (MAPK). However, treatment with NAC (reactive oxygen species scavenger) and MAPK-specific inhibitors (SP600125 and SB203580) could reverse the changes of these apoptotic-related proteins. These results suggested that TFAE possessed potential anticancer activity in HepG2 cells through ROS-mediated mitochondrial dysfunction involving MAPK pathway.

## 1. Introduction

Hepatocellular carcinoma (HCC) is one of the commonest forms of cancer worldwide, especially in China; it accounts for about 55% of total HCC cases [1, 2]. Currently surgical resection or liver transplantation may be the most efficient therapeutic strategy for early stage of HCC, and over 80% patients cannot receive effective treatment due to the failure of conventional therapy [3]. Thus, it is necessary to develop novel drugs for HCC. Recently, researchers pay increasing attention to integrated therapy for HCC, in which natural products play important roles due to their unique advantages [4, 5].

*Arachniodes exilis*, a fern belonging to Arachniodes species (Dryopteridaceae), is widely distributed in the tropical and subtropical moist areas of the world, especially in the eastern and southeastern Asia. In China, this plant is mainly distributed in the south areas of the Yangtze River and Shandong and Henan provinces [6].* A. exilis *has been used as a folk medicine for a long time to treat acute jaundice hepatitis, arthritis, lumbago, dysentery, and burn injuries and has been proved to have antibacterial, anti-inflammatory, and sedative activities by modern pharmacological studies [7]. Studies have suggested that Dryopteridaceae plants usually possess antivirus and anticancer effect, which are based on the bioactive constituents of flavonoids and phloroglucinol derivatives [8]. The anticancer mechanisms of dryopteridaceae plants are different from the common chemotherapeutic drugs. They possess the cytotoxicity to tumor cells, meanwhile, without damage to the hematopoietic stem cells [9].* A. exilis* has been confirmed to contain similar ingredients as other dryopteridaceae plants, which allows us to anticipate the potential antitumor effect of this plant. However, none of the relative study is found in literature.

Apoptosis is a highly regulated process leading to programmed cell death, which is under the control of a number of signaling pathways including caspase and MAPK pathways [10, 11]. Apoptosis has been identified to be induced via two major pathways: the death receptor-induced pathway and the mitochondrial-mediated pathway, in which mitochondria-mediated pathway plays a vital role in the process of apoptosis [12, 13]. HepG2 cell line is the most frequently used experimental model for in vitro researches on HCC [12]. In this study, the antiproliferation effect of the total flavonoids of* A. exilis *(TFAE) on HepG2 cells was confirmed, and the apoptosis induction and the effects on the expression of apoptosis-related proteins were evaluated. Moreover, the underlying molecular mechanisms of anti-HCC activities of TFAE were explored.

## 2. Materials and Methods

### 2.1. Reagents

Dulbecco's modified Eagle's medium (DMEM) was obtained from HyClone Laboratories (Logan, UT, USA). Fetal bovine serum (FBS) was purchased from Trans Gen. Biotech (TRANS, Beijing, China). Trypsin, dimethyl sulfoxide (DMSO), and agarose were obtained from Beijing Solarbio Science & Technology. CCK-8 cell viability assay kit was purchased from Beijing Zoman Biotechnology Co., Ltd. Annexin V-FITC Apoptosis Detection Kit was purchased from Hangzhou MultiSciences Biotech Co., Ltd. ROS detection kit, mitochondrial membrane potential assay kit (JC-1), and Hoechst 33342 staining kit were purchased from Jiangsu Beyotime Institute of Biotechnology. Antibody against caspase-9 was obtained from Proteintech Group (CHI, USA). Antibodies specific for caspase-3, cytochrome c, Bcl-2, Bax, phosphor-ERK, ERK, phosphor-p38, p38, phosphor-JNK, JNK, and actin were purchased from Santa Cruz Biotechnology (CA, USA). All other chemical agents were of analytical grade unless otherwise specified.

### 2.2. Plant Materials

The roots of* A. exilis *were collected from Xinzhi, Jiujiang city, Jiangxi province, China, and authenticated by Professor Ceming Tan of the Wild Plant Herbarium of Jiujiang City Forestry Bureau. A voucher specimen (number F1205) has been deposited in the Department of Pharmacy, Basic Medical School of Jiujiang University.

### 2.3. Preparation of TFAE

Appropriate dried powder (500 g, 20–30 mesh) of* A. exilis* roots was extracted by 60% ethanol (solid-liquid ratio of 1 : 30) with ultrasonic for 3 times, and each time for 2 hours. The extract of* A. exilis* was obtained by concentrating the extracting solution to dryness in a rotary evaporator and a freeze dryer.

The purification process of extract of* A. exilis* was performed in the polyamide column chromatography. Briefly, the extract was dissolved in water, and the well-mixed solution was added into the column. The absorbed polyamide was initially eluted with water to remove extraneous constituents with strong polarity, then with 70% ethanol to increase the amount of flavonoids. The evaporation and freeze-drying of the 70% ethanol eluate yielded TFAE.

### 2.4. Determination of the Total Flavonoids Content in TFAE

The total flavonoids in TFAE were estimated as rutin equivalent. Briefly, a series of rutin solutions in the concentration range of 0–65.0 *μ*g/mL were used for the calibration curve and were prepared as below: 25 mL of rutin samples at certain concentrations was prepared by step by step mixing appropriate volumes of rutin stock solution with 1 mL of 5% (w/v) NaNO_2_ solution, 1 mL of 10% Al(NO_3_)_3_ solution, and 10 mL of a 10% (w/v) NaOH solution, and after each adding, the mixtures were allowed to stand for 6 min. Finally, the mixtures were made up to 25 mL with distilled water and mixed well. The absorbance was measured with a spectrometer (Purkinje General Instrument Co., Ltd, Beijing, China) after 15 min. Similarly, the TFAE sample was prepared and analyzed as above. On the basis of the calibration curve, the total flavonoids content of TFAE was calculated.

### 2.5. Cell Culture and Sample Preparation

Human hepatoma HepG2 cells and normal human liver cells LO2 were kindly provided by the Department of Hepatobiliary Surgery, Tongji Hospital (Wuhan, China) and were maintained in DMEM medium, which was supplemented with 10% FBS at 37°C in 5% CO_2_ humidified atmosphere.

Five milligram of TFAE was dissolved in DMSO and triple-distilled water to make a stock solution of 100 mg/mL (the final DMSO concentration was controlled less than 0.1%). The stock solution was sterilized using a sterile 0.22 *μ*m membrane filter. The working solutions with different concentrations were freshly prepared by diluting appropriate volumes of the stock solution with the basal medium containing FBS.

### 2.6. Assay for Cell Proliferation Activity

CCK-8 assay was employed to detect the effect of TFAE on cell proliferation of HepG2 and LO2 cells. Briefly, both cells from exponentially growing cultures were harvested in 96-well plates (5 × 10^3^/well). After complete adhesion, the cells were treated with TFAE at indicated concentrations for 24 or 48 h. The cells were then stained with CCK-8 (10 *μ*L/well) and incubated for 4 h. The optical density of each well was recorded at 450 nm in a microplate reader (Power-wave XL, Bio-Tek, USA). The IC_50_ values were determined as the concentrations that inhibited cell viability by 50%.

### 2.7. Flow Cytometry Assay for HepG2 Cells

Flow cytometry analysis was performed to detect apoptosis by Annexin V-FITC Apoptosis Detection Kits [14]. Briefly, HepG2 cells were seeded in 6-well plates (5 × 10^5^/well) and treated with TFAE at different concentrations (15.0, 30.0, and 60.0 *μ*g/mL) for 24 h. Both adherent and floating cells were collected and washed twice with phosphate-buffered saline (PBS). All cells were resuspended in 500 *μ*L binding buffer and incubated with 5 *μ*L FITC-conjugated Annexin V and 10 *μ*L propidium iodide (PI) for 10 min at room temperature in the dark. Finally, the cells were analyzed with a FACSort flow-cytometer (Becton Dickinson and Company, USA).

### 2.8. Fluorescent Staining of Nuclei for HepG2 Cells

HepG2 cells from exponentially growing cultures were seeded in 24-well plates (2 × 10^4^/well) and incubated with TFAE (controlled the final concentrations of TFAE at 15.0, 30.0, and 60.0 *μ*g/mL) for 24 h. After the treatment, the cells were washed with PBS and fixed in MeOH-HAc (3 : 1, v/v) for 10 min at 37°C. The cells were stained with Hoechst 33342 (10 *μ*g/mL in PBS) in dark for 10 min at room temperature and then examined in a fluorescence microscope (Olympus, Tokyo, Japan) at 340 nm.

### 2.9. Measurement of Intracellular Reactive Oxygen Species (ROS)

The intracellular ROS was measured with an oxidant sensitive fluorescent probe dichlorofluorescein-diacetate (DCFH-DA). Briefly, HepG2 cells were seeded in 6-well plates (5 × 10^5^/well) and treated with TFAE at the indicated concentration for 4, 12, and 24 h. After that, the cells were harvested and stained with 10 *μ*M DCFH-DA for 30 min at 37°C in the dark. The intracellular ROS production was detected by the flow cytometric assay.

### 2.10. Measurement of Mitochondrial Membrane Potential (ΔΨ*m*)

HepG2 cells from exponentially growing cultures were seeded in 6-well plates (5 × 10^5^/well). After treatment of HepG2 cells with 30.0 *μ*g/mL of TFAE for 4, 12, and 24 h, the cells were harvested and stained with JC-1 working solution for 20 min at 37°C in the dark. After being washed twice with the JC-1 binding buffer, the fluorochrome-conjugated cells were analyzed by flow cytometric assay.

### 2.11. Determination of the Expression Levels of Apoptosis- and MAPK-Related Proteins

HepG2 cells were treated with the indicated concentration of TFAE for a given period of time and then harvested with ice-cold PBS. The protein samples were prepared according to the description of the literature [14]. After the determination of protein contents via the Bio-Rad protein assay, these protein samples (40–50 *μ*g) were separated by 12% SDS-PAGE and electrotransferred to nitrocellulose membrane. The membranes were blocked with 5% (w/v) nonfat dry milk for 1 h and incubated with primary antibodies cytochrome c, cleaved caspase-3, cleaved caspase-9, PARP, Bcl-2, Bax, ERK, P-ERK, JNK, P-JNK, p38, and P-p38 for 2 h at room temperature. After washing, the membranes were developed by the corresponding secondary antibodies and visualized by enhanced chemiluminescence procedures (Beyotime, Haimen, China) according to the manufacturer's instructions. Densitometric analysis of immunoblots was performed with AlphaEaseFC software. The expression of actin was used as a control.

### 2.12. Inhibitor Treatment

To clarify the roles of ROS and MAPK inhibitors on TFAE-induced apoptosis in HepG2 cells, the cells were pretreated for 2 h with the following inhibitors individually before the treatment of TFAE (30.0 *μ*g/mL): 5 mM N-acetyl cysteine (NAC), 20 *μ*M anthrapyrazolone (SP600125, a JNK-specific inhibitor), or 20 *μ*M hydrochloride4-(4-fluorophenyl)-2-(4-methylsulfinylphenyl)-5-(4-pyridyl) 1 H-imidazole (SB203580, a p38-specific inhibitor).

### 2.13. Statistical Analysis

For all parameters measured, the values for all samples in triplicate experimental conditions were averaged, and the SD of the mean was calculated. All data were analyzed using SPSS 16.0 software (SPSS, USA). Statistical significance was assessed by one-way ANOVA. Differences with *P* < 0.05 were considered to be statistically significant.

## 3. Results

### 3.1. Determination of the Total Flavonoids Content in TFAE

The detection wavelength was 486.6 nm based on the spectral scanning of rutin and the regression equation of the calibration curve of rutin was *Y* = 82.645*X* + 2.661 (*R* ^2^ = 0.9998, where *Y* is the absorbance, *X* is the concentration (*μ*g/mL), 82.645 is the slope, and 2.661 is the *Y*-intercept). The content of the total flavonoids in extract was 56.4% before the purification process, and then up to 82.9% after the polyamide purification process.

### 3.2. Effect of TFAE on Cell Proliferation Activity

**Figure 1:**
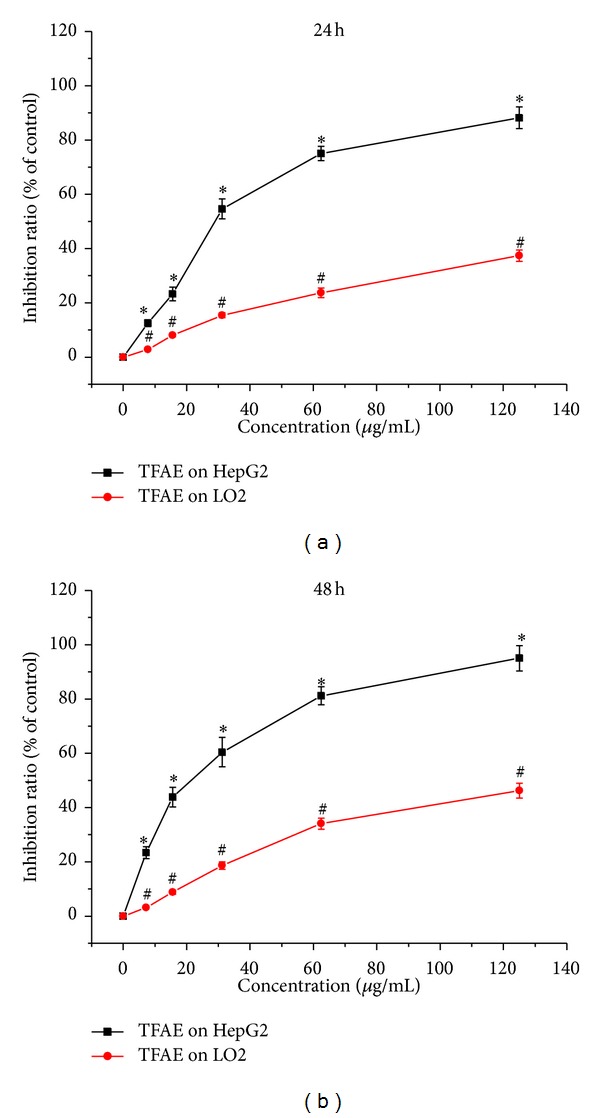
The effect of TFAE on cell proliferation inhibition in HepG2 and LO2 cells for 24 or 48 h by CCK-8 assay. *P < 0.05, TFAE-treated group compared with the negative control group. ^#^ *P* < 0.05, the inhibition ratio of TFAE-treated LO2 cells group compared with TFAE-treated HepG2 cells group.

The effect of TFAE on cell proliferation of HepG2 and LO2 was determined by CCK-8 assay and the results are shown in Figure 1. It was found that the cell proliferation of HepG2 was obviously inhibited and the inhibition effect of TFAE on HepG2 cells was in a dosage-dependent manner. Compared with the negative control group, the difference was statistically significant (*P* < 0.05). After HepG2 cells were exposed with TFAE for 24 or 48 h, the calculated IC_50_ values were 31.2 and 26.0 *μ*g/mL, respectively. However, as for normal cells of LO2, the IC_50_ values were 225.1 and 133.3 *μ*g/mL, respectively, corresponding to TFAE exposure for 24 and 48 h. These results indicated that TFAE could obviously inhibit the viability of HepG2 cells and showed much less cytotoxicity to LO2 cells.

### 3.3. Effect of TFAE on Apoptosis in HepG2 Cells

**Figure 2:**
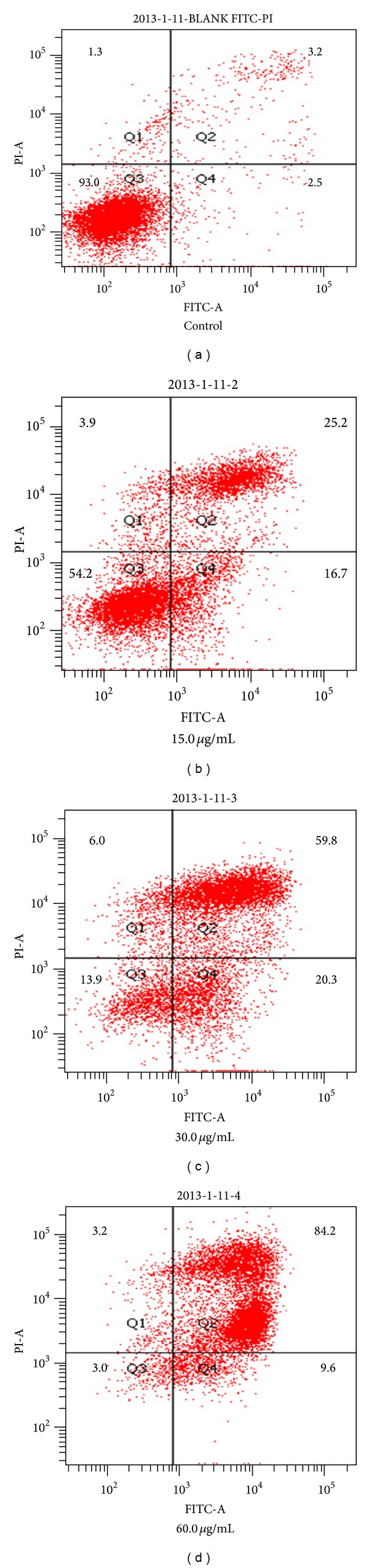
The effect of TFAE on the apoptosis in HepG2 cells for 24 h. The cells were double stained with Annexin V-FITC/PI and analyzed by flow cytometry. The apoptotic ratio of HepG2 cells was expressed as % of total cells.

To explore the effect of TFAE on the apoptosis in HepG2 cells, flow cytometry assay was performed by double staining with FITC-conjugated Annexin V and PI. The ratios of normal cells, early apoptotic cells, and late apoptotic cells are shown in Figure 2. HepG2 cells showed that various degrees of apoptosis with TFAE concentration gradually increased. As for the early apoptotic cells, they can only be stained by FITC and are expressed in the lower right quadrant scatter of the pictures. The early apoptosis ratios of HepG2 cells were 16.7, 20.3, and 9.6%, individually corresponding to 15.0, 30.0, and 60.0 *μ*g/mL of TFAE treatment for 24 h. As for late apoptotic cells, they can be stained by both FITC and PI and were expressed in the upper right quadrant scatter of the pictures. The late apoptosis ratios of HepG2 cells were 25.2, 59.8, and 84.2%, individually corresponding to 15.0, 30.0, and 60.0 *μ*g/mL of TFAE treatment for 24 h. The results indicated that appropriate concentration of TFAE induced apoptosis in HepG2 cells.

**Figure 3:**
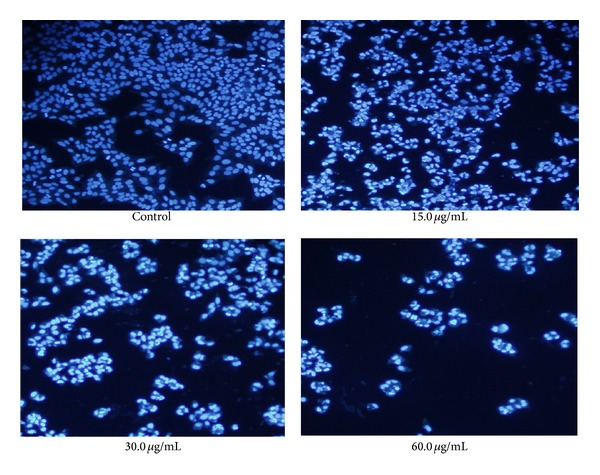
Morphological effects of TFAE on HepG2 cells with Hoechst 33342 fluorescence assay.

To further analyze the apoptosis in HepG2 cells, fluorescence staining of nuclei with Hoechst 33342 was used for the examination of the chromatin condensation and apoptotic bodies. As shown in Figure 3, HepG2 cells for the control group were able to grow well as normal; the cell nucleus and chromatin showed weak light blue fluorescent in uniform dispersion, and the cell nucleus exhibited round or oval nuclei. In contrast, HepG2 cells treated with different concentrations of TFAE presented different degrees of cell growth inhibition like cell shrinkage, cell size decrease, karyopyknosis, and chromatin margination. And meanwhile, a series of typical apoptotic morphology including apoptosis bodies and nuclear fragments were observed.

### 3.4. Effect of TFAE on Intracellular ROS and Mitochondrial Membrane Potential (ΔΨ*m*) in HepG2 Cells

**Figure 4:**
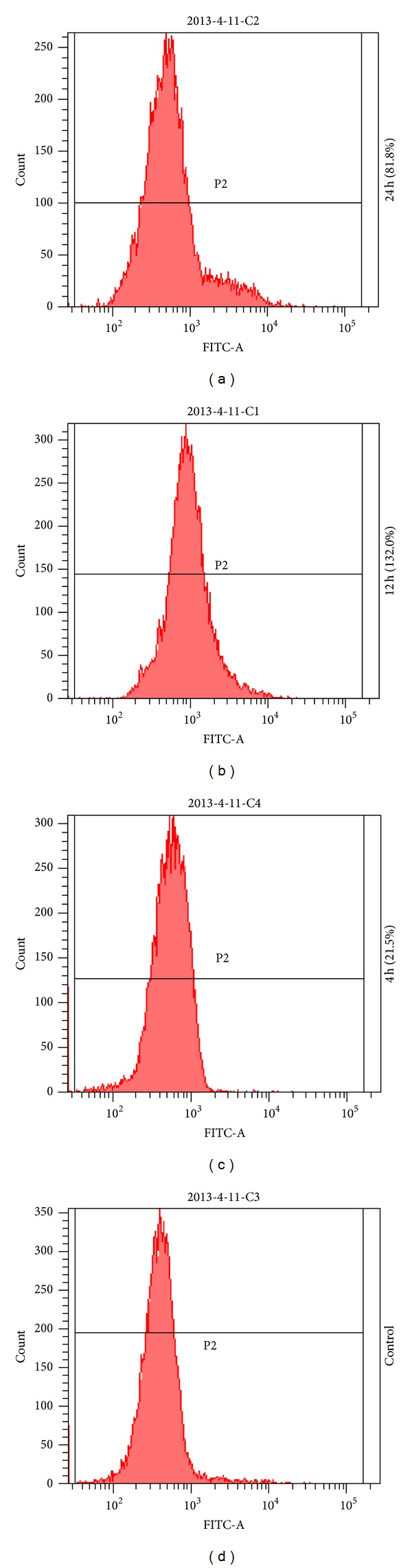
The effect of TFAE (30.0 *μ*g/mL) on reactive oxygen species in HepG2 cells. Time-dependent change of reactive oxygen species stained with DCFH-DA and analyzed by flow cytometry.

Compared with the control group, HepG2 cells treated with 30.0 *μ*g/mL TFAE could lead to the increase of fluorescence intensity, indicating the increase of intracellular ROS. With TFAE treating time prolonging, the levels of intracellular ROS firstly increased gradually and reached a peak value at 12 h. And then the ROS intracellular levels declined gradually. But the intracellular DCF fluorescence intensity remained at remarkably higher levels than the control group even after 24 h. As shown in Figure 4, when compared with the control group, after treatment with TFAE for 4, 12, and 24 h, the mean intracellular DCF fluorescence intensities were increased 21.5, 132.0, and 81.8%, respectively. The results showed that TFAE induced increased level of intracellular ROS in HepG2 cells and eventually caused the death of cells.

**Figure 5:**
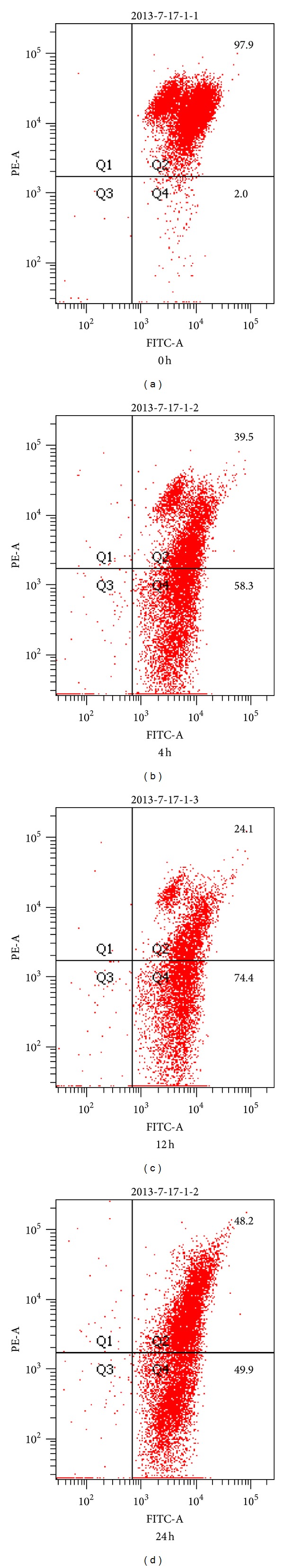
The effect of TFAE (30.0 *μ*g/mL) on mitochondrial function in HepG2 cells. Time-dependent change of ΔΨ*m* stained with JC-1 and analyzed by flow cytometry.

As shown in Figure 5, the mitochondrial membrane potential in HepG2 cells reduced gradually and touched the bottom after 12 h when treated with TFAE. The ratios of cells with green light were 58.3, 74.4, and 49.9% when HepG2 cells were treated with 30.0 *μ*g/mL TFAE for 4, 12, and 24 h, respectively. The obtained results showed that TFAE could cause mitochondrial dysfunction of HepG2 cells, indicating the possible role of TFAE by inducing apoptosis in HepG2 cells through the mitochondrial pathway.

### 3.5. Effect of TFAE on the Expression of Apoptosis- and MAPK-Related Proteins

**Figure 6:**
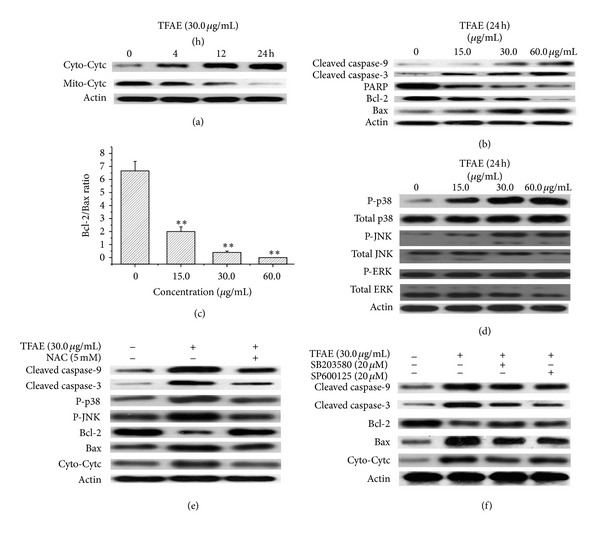
The effect of TFAE on the expression of apoptosis-related proteins in HepG2 cells by Western blot analysis (a) cytochrome c release from mitochondria into cytosol; (b) activation of caspase family members as well as modulation of Bcl-2 family members; (c) reduction of the ratio of Bcl-2/Bax, **P < 0.01 versus the control group; (d) TFAE-induced phosphorylation of MAPK pathway; (e) effect of NAC on TFAE-induced apoptosis and the phosphorylation of JNK and p38; (f) effects of SB203580 and SP600125 on the TFAE-induced apoptosis.

In the apoptosis induction process of mitochondrial pathway, the early important feature is the releasing process of cytochrome c from the mitochondria. As shown in Figure 6(a), when HepG2 cells were treated with 30.0 *μ*g/mL TFAE for 4, 12, and 24 h, cytochrome c was observed to be released from the mitochondria and then entered into the cytosol, showing a significant time-dependent manner. Compared with the control group, the difference of the protein expression level was statistically significant.

The activation of caspase family members is considered as the best hallmark of apoptosis. As shown in Figure 6(b), after HepG2 cells were treated with 15.0, 30.0, and 60.0 *μ*g/mL of TFAE for 24 h, the expression level of cleaved caspases-3 and -9 was remarkably increased and meanwhile PARP was hydrolyzed. Accordingly, compared with the control group, the expression level of proapoptotic protein Bax increased significantly accompanied with the significant reduction of the expression level of antiapoptotic protein Bcl-2 for all the tested groups. Figure 6(c) shows that the ratio of Bcl-2/Bax was significantly decreased in a dose-dependent manner.

The levels of MAPKs like JNK, ERK, and p38 were investigated to clarify the involvement of MAPKs in TFAE-induced apoptosis in HepG2 cells. As shown in Figure 6(d), when compared with the control group, the phosphorylation of JNK and p38 was significantly increased with the increase of TFAE, and meanwhile, the expression of total JNK and total p38 had no significant difference. However, the expression of phosphorylated ERK and total ERK had no significant difference.

To further clarify the possible role of ROS in TFAE-induced apoptosis, HepG2 cells were incubated with 5 mM NAC and treated with 30.0 *μ*g/mL TFAE for 24 h. After the treatment, the values of cleaved caspases-3, -9, Bcl-2, Bax, P-JNK, P-p38, and cytochrome c were detected. As shown in Figure 6(e), NAC, as a capturing agent of ROS, could significantly reverse the expression of related proteins of TFAE-induced apoptosis in HepG2 cells.

To further confirm the possible role of MAPK pathway in the process of apoptosis in HepG2 cells, SB203580 (p38 specific inhibitor) and SP600125 (JNK specific inhibitor) were used to pretreat HepG2 cells for 2 h and then treated with 30.0 *μ*g/mL TFAE for 24 h, respectively. As shown in Figure 6(f), SP600125 and SB203580 could significantly reverse the expression of related proteins of TFAE-induced apoptosis in HepG2 cells.

## 4. Discussion

In recent years, the development of anticancer drugs is very quick. Many natural substances with low toxicity and cost are acted as irreplaceable roles in lead compounds exploration, attracting increasing attention in this field [4]. The main ingredients in* A. exilis* are flavonoids and phenols including flavonones (eriodictyol, miscanthoside, and eriocitrin), flavones (cyanidenon), and flavan-3,4-diols (arachniodesin A, arachniodesin B, and epicatechin) [15, 16]. Many studies proved that flavonoids can induce the cell apoptosis like colon cancer, liver cancer, ovarian cancer, and breast cancer [17, 18].

The study found that TFAE could significantly inhibit the proliferation of human hepatoma HepG2 cells in a dosage- and time-dependent manner, whereas it showed much less cytotoxicity to normal LO2 cells. We speculate that this phenomenon is associated with cellular metabolic pathways. The metabolic pathway of liver LO2 cell is aerobic oxidation pathway, while the metabolic pathway of tumor cells is glycolysis pathway. Existing literature confirmed that flavonoids can inhibit glycolytic enzymes (such as HIF-1*α*) activity that affect the metabolism of tumor cells and promote apoptosis of tumor cells [19]. Since the metabolism of normal liver cells is by way of aerobic oxidation, changes in glycolytic enzymes will have less effect on LO2 cells' metabolization. ERK/MAPK signal transduction pathway can regulate the expression of HIF-1*α*, and the expression of HIF-1*α* may regulate the expression of target gene associated with tumor [20, 21]. However, the exact reason that TFAE exhibited stronger apoptotic effect on HepG2 cells than normal liver LO2 cell line requires further experimental confirmation.

Compared with the control group, the apoptosis ratio of HepG2 cells increased gradually with the concentration increase of TFAE, showing a clear dosage-dependent manner. Meanwhile, mitochondrial membrane potential was significantly decreased during the treatment of TFAE, which suggested that TFAE could cause mitochondrial dysfunction of HepG2 cells and possibly induce apoptosis in HepG2 cells through mitochondrial pathway [22]. In the process of the TFAE-inducted apoptosis in HepG2 cell, the levels of ROS increased significantly, confirming that the oxidative stress-induced apoptosis played a crucial role in TFAE-induced apoptosis. As we all know that active oxygen is a class of reactive oxygenated substances produced in the process of aerobic metabolism of the body, including superoxide anion radicals, hydroxyl radicals, and hydrogen peroxide. Excessive active oxygen can cause cell toxicity, cell apoptosis, and body injury and eventually leading to cell death [23, 24]. For the mitochondrial, ROS can lead to the openness of PT pore and can further trigger the release of contents of mitochondrial. Finally, it can result in a vicious cycle of amplification effect [25].

Apoptosis is a programmed cell death regulated by multiple genes. The family members of Bcl-2 constitute a complex network to participate in the regulation of apoptosis and become a target of treating tumors [26]. Bcl-2 family includes antiapoptotic genes (such as Bcl-2 and Bcl-xL) and proapoptotic genes (such as Bax, Bad, and Bak), and genes of Bax and Bcl-2 have 40% homology [27, 28]. When the ratio of Bax/Bcl-2 is high, the cells apoptosis tend to increase; otherwise the cells apoptosis will decrease [29]. The results of Western blot showed that, after HepG2 cells were treated with TFAE, the expression of antiapoptotic protein Bcl-2 decreased, whereas the expression of proapoptotic protein Bax and the ratio of Bax/Bcl-2 increased. Therefore, it can be concluded that Bcl-2 family proteins were involved in the regulation of TFAE-inducted apoptosis in HepG2 cells through the mitochondrial pathway.

The activation of caspase enzymes is the important biochemical change of apoptosis [30, 31]. The activation of caspase-9 can promote the activation of caspase-3, and caspase-3 plays a vital role in the apoptotic process [32, 33]. The experimental results showed that the activation of cleaved caspases-3 and -9 was caused by TFAE in a dosage-dependent manner, indicating the caspase pathways were involved in the process of TFAE-induced apoptosis in HepG2 cells.

MAPK pathway is an important intracellular signal transduction pathway and plays an important role in the development of tumor. MAPK has four major subfamilies including ERK, JNK, p38 MAPK, and ERK5 [34]. Among those, JNK and p38 MAPK play important roles in apoptosis stress reaction [35, 36]. ERK regulates cell growth and differentiation [37]. The results of Western blot analysis showed that TFAE could induce the significant increase of the expression of phosphorylated JNK and phosphorylated p38 in a dosage-dependent manner, and meanwhile the expression of total JNK and total p38 has no significant difference. However, the expression of phosphorylated ERK and total ERK had no significant difference compared with the control group. Therefore, it can be concluded that the signal transduction pathways of JNK and p38 MAPK may be involved in the process of TFAE-induced apoptosis in HepG2 cells.

As known to all, low level of ROS can promote cell proliferation to some extent; relatively high level of ROS can induce cells apoptosis, and even higher level of ROS might directly cause cell necrosis [38]. In continuing oxidative stress effect of ROS, tumor cells with higher level of ROS tend to die easier than normal cells. Therefore, using the feature of the tumor cells highly sensitive to ROS to explore new antitumor drugs may get new clinical drugs with low toxicity and good efficacy [39, 40]. In the present study, the production of intracellular ROS was significantly increased in a time-dependent manner and reached the peak value when treated with TFAE for 12 h, which demonstrated that TFAE could lead to the accumulation of intracellular ROS.

To further clarify the relationship between ROS and TFAE-induced apoptosis in HepG2 cells, the cells were pretreated with NAC before the treatment of TFAE. It was found that the related values of cleaved caspase-3, -9, Bcl-2, Bax, P-JNK, P-p38, and cytochrome c were changed when compared with the experiment results without NAC, demonstrating that NAC (as a capturing agent of ROS) could significantly reverse TFAE-induced apoptosis in HepG2 cells and hereby confirm the role of ROS in the process of TFAE-induced apoptosis in HepG2 cells.

To further confirm the possible role of MAPK pathway in the process of apoptosis in HepG2 cells, SB203580 and SP600125 were used to pretreat HepG2 cells before the treatment of TFAE. It was found that SP600125 and SB203580 could significantly reverse TFAE-induced apoptosis in HepG2 cells, further confirming the important role of p38 and JNK in the process of TFAE-induced apoptosis in HepG2 cells.

In summary, TFAE significantly inhibited the proliferation of hepatoma cells HepG2 through apoptotic pathway. TFAE caused the accumulation of ROS in the cells, resulting in the regulation of JNK and p38 MAPK, and ultimately activated the downstream molecular events of mitochondrial apoptosis pathway, such as the activation of caspases-3 and -9 and the modulation of Bcl-2 family members. As a conclusion, TFAE showed great potential to be developed as a new anticancer drug with low toxicity and good efficacy.
